# Geological duration of ammonoids controlled their geographical range of fossil distribution

**DOI:** 10.7717/peerj.4108

**Published:** 2017-11-28

**Authors:** Ryoji Wani

**Affiliations:** Faculty of Environment and Information Sciences, Yokohama National University, Yokohama, Japan

**Keywords:** Ammonoidea, Hatchling size, Reproductive strategy, Latitudinal distribution, Habitat, Geological duration

## Abstract

The latitudinal distributions in Devonian–Cretaceous ammonoids were analyzed at the genus level, and were compared with the hatchling sizes (i.e., ammonitella diameters) and the geological durations. The results show that (1) length of temporal ranges of ammonoids effected broader ranges of fossil distribution and paleobiogeography of ammonoids, and (2) the hatchling size was not related to the geographical range of fossil distribution of ammonoids. Reducing the influence of geological duration in this analysis implies that hatchling size was one of the controlling factors that determined the distribution of ammonoid habitats at any given period in time: ammonoids with smaller hatchling sizes tended to have broader ammonoid habitat ranges. These relationships were somewhat blurred in the Devonian, Carboniferous, Triassic, and Jurassic, which is possibly due to (1) the course of development of a reproductive strategy with smaller hatchling sizes in the Devonian and (2) the high origination rates after the mass extinction events.

## Introduction

**Figure 1:**
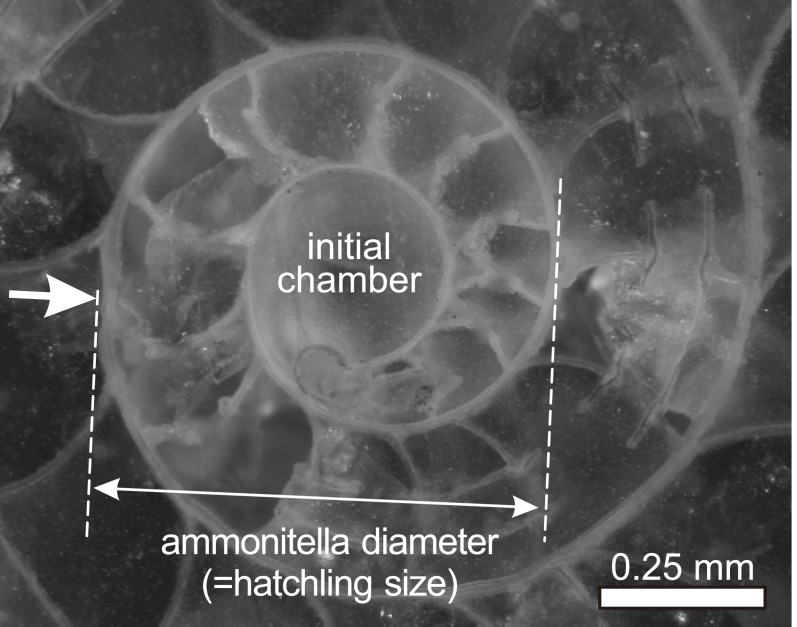
Ammonitella diameter in ammonoids. Photograph of embryonic shell of Desmoceras latidorsatum from Cretaceous. Arrow indicates the primary constriction.

Reproductive strategy is one of the major factors controlling the geographic distribution, evolutionary and extinction rates, and speciation in marine animals (Yacobucci, 2016 for ammonoids). Yacobucci (2016) presented a model for ammonoid speciation, based on their evolutionary characteristics, including heterochrony, homeomorphy, and a high origination rate that is often linked to sea level cycles. In modern cephalopods (squids, cuttlefishes, octopuses, and nautiluses), Villanueva et al. (2016) analyzed the hatchling sizes and their geographical ranges, and suggested that species of smaller hatchling size with a planktic post-embryonic mode of life have broader geographical ranges. In ammonoids, embryonic shells can be recognized by the presence of a primary constriction in the innermost whorl (Landman, Tanabe & Shigeta, 1996; De Baets, Landman & Tanabe, 2015; and references therein). The embryonic ammonoid, which is termed ammonitella (Drushchits & Khiami, 1970), consists of an initial chamber and about one planispiral whorl from the caecum terminating at the primary constriction (Fig. 1). Most ammonoid hatchlings are thought to have had a planktic mode of life (Kulicki, 1974; Kulicki, 1979; Kulicki, 1996; Drushchits, Doguzhayeva & Mikhaylova, 1977; Tanabe, Fukuda & Obata, 1980; Tanabe et al., 2001; Tanabe, Landman & Yoshioka, 2003; Landman, 1985; Tanabe & Ohtsuka, 1985; Landman, Tanabe & Shigeta, 1996; Westermann, 1996; Rouget & Neige, 2001; Ritterbush et al., 2014; De Baets, Landman & Tanabe, 2015). Therefore, a planktic mode of life at post-embryonic stages in ammonoids is expected to have had a significant impact on their geographical range (Landman, Tanabe & Shigeta, 1996; Westermann, 1996; Tajika & Wani, 2011; Ikeda & Wani, 2012; Brayard & Escarguel, 2013; Yahada & Wani, 2013; Zacaï et al., 2016). However, there has been little quantitative analysis on how hatchling size related to their geographical ranges.

The purpose of this study is to evaluate the relationship between hatchling size and geographical ranges in ammonoids. Second, this study evaluates the relationship between geological duration and geographical ranges in ammonoids. This is of value because the geographical ranges of fossil marine organisms would appear to be positively correlated with geological duration (Jablonski, 1987; Miller, 1997; Liow, 2007; Foote et al., 2008). Three parameters for each species were chosen in order to examine the possible influence of hatchling size and geological duration on the geographic distribution of ammonoids, which would provide new insights into understanding the early life history and reproductive strategy in ammonoids: mean hatchling size, latitudinal geographical range, and geological duration. The former two parameters are similar to those used by Villanueva et al. (2016) for the analyses of modern cephalopods.

## Materials and Methods

This study analyzed the relationship between latitudinal distributions and hatchling sizes in Devonian–Cretaceous ammonoids at the genus level only since the examined data set is available at this taxonomic level. A total of 223 genera (30 genera from the Devonian, 28 genera from the Carboniferous, 25 genera from the Permian, 55 genera from the Triassic, 24 genera from the Jurassic, 61 genera from the Cretaceous) were analyzed for this study. The hatchling sizes (i.e., ammonitella diameters; Fig. 1) of the examined genera were obtained from De Baets, Landman & Tanabe (2015) (Table S1). The mean ammonitella diameter for each genus was calculated from the data of ammonitella diameters of the species of the same genus. The data of ammonitella diameters were grouped and analyzed for the aggregate Devonian–Cretaceous interval and for each constituent period (Devonian to Cretaceous).

For each ammonoid genus, the geographic extent of their fossil occurrences was obtained through literature reviews (Chlupáč & Turek, 1983; Korn & Klug, 2002; Korn & Ilg, 2007; De Baets, Klug & Korn, 2009, for the Devonian; Korn & Ilg, 2007; Furnish et al., 2009, for the Carboniferous and Permian; Arkell et al., 1957; Tozer, 1981, ET Tozer, pers. comm., 1989, for the Triassic; Arkell et al., 1957; Howarth, 2013; Hoffmann, 2015, for the Jurassic; Arkell et al., 1957; Wright, Calloman & Howarth, 1996; Igolnikov, 2007, for the Cretaceous). The paleogeographic maps for each period (Scotese, 2011) were used to calculate the latitudinal ranges of the examined ammonoids. In this study, the geographical distribution of the examined genera was evaluated by latitudinal ranges, which is a similar approach taken by the analysis of modern cephalopods (Villanueva et al., 2016). The latitudinal ranges were transformed into distance (km) by applying the Haversine formula to calculate the great-circle distance between two points (mean radius of the Earth = 6,371 km):

\begin{eqnarray*}2\pi \times (\text{mean radius of the Earth})\times \frac{\text{latitudinal range}(\textdegree )}{360} . \end{eqnarray*}

The latitudinal distribution of the examined genera were analyzed with respect to the geological duration of each genus. The geological durations were obtained from the geological ages at the stage level that were adapted from those in Gradstein et al. (2012). This is because the examined data set is available at this stage level. In the case that the geological durations were significantly correlated to the latitudinal ranges of ammonoids, an index is introduced in order to reduce the influence from the geological durations of the examined ammonoids: (latitudinal range)/(geological duration). This index corresponds to the latitudinal ranges per unit of time. This index was calculated for each genus and was compared to their ammonitella diameters.

Values were compared using analysis of variance (ANOVA) for the regression analyses and differences were considered significant at *P* < 0.05. Linear regressions (reduced major axes) were used for the graphics. The linear regressions with ANOVA are one of the most simple and basic approaches, so that this study aims to recognize principal relationships among the examined parameters. Linear regressions are used in similar analyses of modern cephalopods in Villanueva et al. (2016), thus the results of this study could be directly comparable to those in Villanueva et al. (2016).

Fossil occurrences might be incomplete and therefore latitudinal distribution based on them as well. Species diversity among fossil invertebrates during the Phanerozoic is known to be highly correlated with volume and area of sedimentary rocks (Raup, 1976). However, such difference of volume and area of sedimentary rocks in examined periods were not considered in this study. Furthermore, it is widely acknowledged that the analysis of comparative data from related species should be performed taking into account their phylogenetic relationships (Paradis & Claude, 2002). Such phylogenetic relationships were not taken into account in this study, because the numbers of the examined genera are not sufficient and the phylogenetic relationships in most examined genera are not clearly recognized.

The 223 genera in this study represent approximately 10% of the number of genera listed in the Paleobiology Database data (approximately 2,500 genera). Analyses of modern cephalopods including approximately 13% of the total number of living cephalopod species described to date (110 species of the 845 living cephalopod species), reveal a positive correlation between the hatchling sizes and geographical ranges (Villanueva et al., 2016). This percentage is comparable to that found in this study.

## Results

### Relationship between latitudinal range and ammonitella diameter

**Figure 2:**
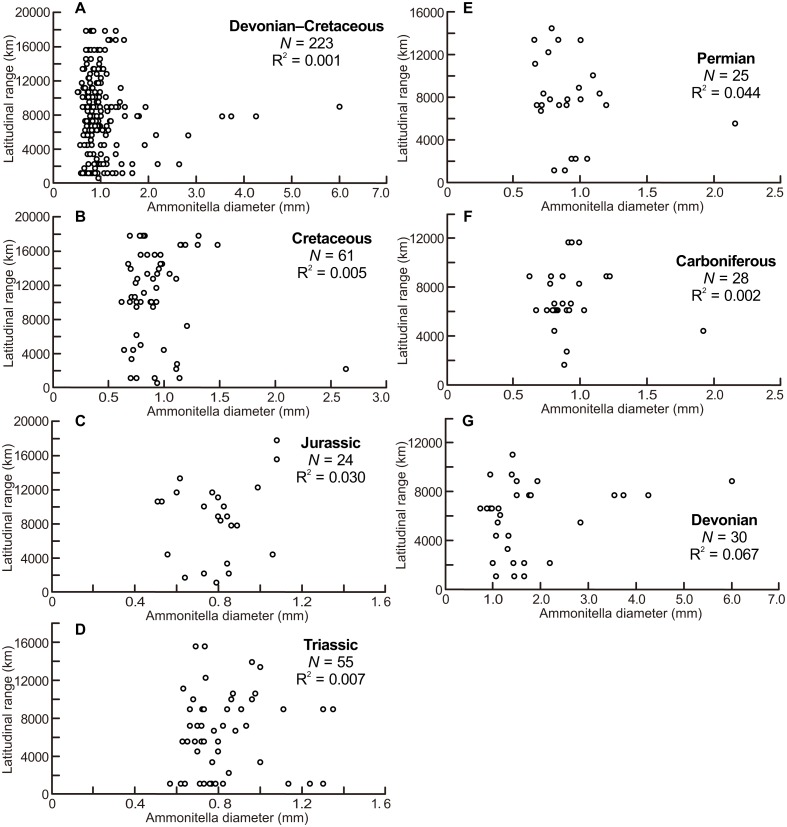
Scatter diagrams between latitudinal ranges and ammonitella diameters. (A) The aggregate Devonian–Cretaceous interval; (B) Cretaceous; (C) Jurassic; (D) Triassic; (E) Permian; (F) Carboniferous; (G) Devonian.

The scatter diagrams for the aggregate Devonian–Cretaceous interval and each constituent period are shown in Fig. 2, which are derived from data summarized in Table 1. No relationship between latitudinal ranges and ammonitella diameters was found for the aggregate Devonian–Cretaceous interval nor for any constituent period (ANOVA, *F* = 0.309 and *P* = 0.58 Devonian–Cretaceous interval, *F* = 2.015 and *P* = 0.17 for the Devonian, *F* = 0.055 and *P* = 0.82 for the Carboniferous, *F* = 1.065 and *P* = 0.31 for the Permian, *F* = 0.354 and *P* = 0.55 for the Triassic, *F* = 0.691 and *P* = 0.41 for the Jurassic, *F* = 0.267 and *P* = 0.61 for the Cretaceous).

**Table 1:** Statistics for examined values of genera. The data of examined genera for aggregate Devonian–Cretaceous interval and each constituent period are summarized.

	No. of genus	Ammonitella diameter (mm)					Latitudinal range (km)				
		Mean	Median	Standard deviation	Min.	Max.	Mean	Median	Standard deviation	Min.	Max.
Devonian–Cretaceous	223	0.998	0.850	0.575	0.51	6.00	7,919	7,780	4,666	556	17,782
Cretaceous	61	0.921	0.850	0.288	0.62	2.64	10,986	11,114	5,311	556	17,782
Jurassic	24	0.794	0.805	0.163	0.51	1.08	8,243	8,891	4,625	1,111	17,782
Triassic	55	0.819	0.760	0.181	0.57	1.35	5,981	5,557	4,237	1,111	15,559
Permian	25	0.922	0.880	0.299	0.66	2.16	7,757	7,780	3,895	1,111	14,448
Carboniferous	28	0.916	0.875	0.236	0.62	1.92	7,026	6,113	2,397	1,667	11,670
Devonian	30	1.787	1.421	1.178	0.73	6.00	5,946	6,668	2,860	1,111	11,114

	No. of enus	Geological duration (My)					Index (latitudinal range/geological duration) (km/My)				
		mean	median	standard deviation	min.	max.	mean	median	standard deviation	min.	max.
Devonian– Cretaceous	223	15.69	12.40	13.46	2.0	67.9	856.14	501.38	982.26	41.62	5,556.93
Cretaceous	61	23.79	17.80	16.92	3.1	67.9	632.47	458.49	511.76	62.44	2,710.70
Jurassic	24	8.55	7.65	12.55	2.0	65.4	1,676.82	1,092.85	1,540.59	261.50	5,556.93
Triassic	55	7.53	4.50	8.33	2.2	37.6	1,308.00	715.25	1,314.86	88.67	5,556.93
Permian	25	23.17	26.60	10.48	3.5	41.9	421.85	318.30	392.45	41.62	2,102.62
Carboniferous	28	15.88	13.60	8.71	7.7	35.4	549.23	462.02	287.67	182.94	1,111.39
Devonian	30	13.48	13.30	4.30	5.0	28.8	474.33	501.38	289.54	77.72	1,587.69

### Relationship between latitudinal range and geological duration

**Figure 3:**
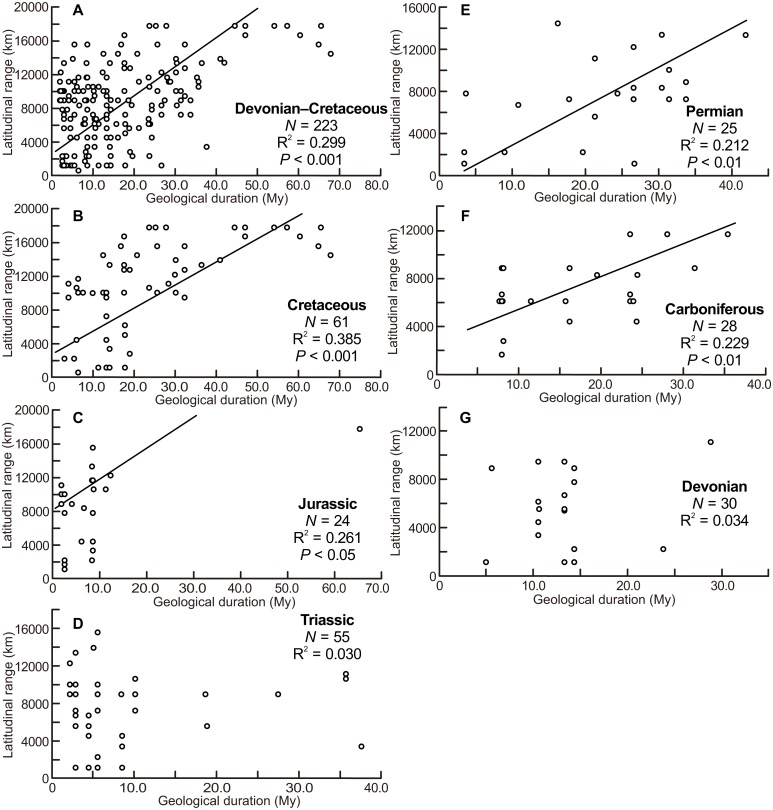
Scatter diagrams between latitudinal ranges and geological durations of genera. (A) The aggregate Devonian–Cretaceous interval; (B) Cretaceous; (C) Jurassic; (D) Triassic; (E) Permian; (F) Carboniferous; (G) Devonian. Regression lines are shown where correlations are statistically significant.

The scatter diagrams for the aggregate Devonian–Cretaceous interval and each constituent period are shown in Fig. 3. Statistically significant relationships between latitudinal ranges and geological durations were found (ANOVA, *F* = 94.340 and *P* < 0.001 for the Devonian–Cretaceous interval, *F* = 7.739 and *P* < 0.01 for the Carboniferous, *F* = 8.148 and *P* < 0.01 for the Permian, *F* = 7.785 and *P* < 0.05 for the Jurassic, *F* = 36.976 and *P* < 0.001 for the Cretaceous). No significant correlation was found in the Devonian (*F* = 0.988 and *P* = 0.33) and Triassic (*F* = 1.624 and *P* = 0.21).

### Relationship between ammonitella diameter and index of latitudinal range and geological duration

**Figure 4:**
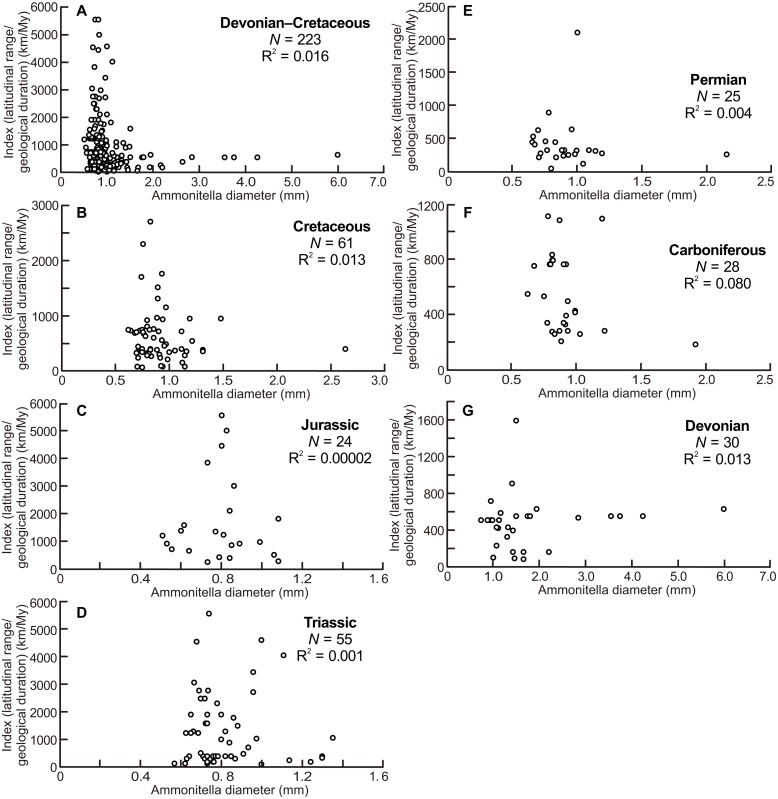
Scatter diagrams between index of latitudinal ranges and geological durations and ammonitella diameters. (A) The aggregate Devonian–Cretaceous interval; (B) Cretaceous; (C) Jurassic; (D) Triassic; (E) Permian; (F) Carboniferous; (G) Devonian.

The scatter diagrams for the aggregate Devonian–Cretaceous interval and each constituent period are shown in Fig. 4. Statistically significant relationships between ammonitella diameter and the index of latitudinal ranges and geological durations were not found (*P* = 0.06, 0.54, 0.14, 0.76, 0.79, 0.98, and 0.37, for the aggregate Devonian–Cretaceous interval, Devonian, Carboniferous, Permian, Triassic, Jurassic, and Cretaceous, respectively). However, the larger indices are observed only in the smaller ammonitella diameters. In the Carboniferous, Triassic, and Jurassic, the larger indices tend to shift to the middle of the observed ranges of ammonitella diameters (Figs. 4C, 4D and 4F).

## Discussion

### Controlling factors for latitudinal ranges of ammonoids

The scatter diagrams between latitudinal ranges and ammonitella diameters (Fig. 2) revealed no relationship between the chosen parameters. In contrast, the hatchling sizes of modern cephalopods together with developmental modes (planktic or benthic) influence the geographical range (Villanueva et al., 2016). The evolutionary history of ammonoids is long (Devonian–Cretaceous), thus the geographical ranges of ammonoids can be considered as an accumulation of multiple geological time slices. Latitudinal ranges of ammonoids had a statistically significant positive correlation with the geological durations, except for the Devonian and Triassic (Fig. 3). These relationships show that the longer the geological duration of taxa, the broader the latitudinal ranges through ammonoid evolutionary history. Such a positive relationship is similar to those in other fossil marine organisms (e.g., gastropods, bivalves, arthropods, foraminifers, radiolarians, crinoids; Jablonski, 1987; Miller, 1997; Liow, 2007; Foote et al., 2008). Evolutionary longevity of ammonoid taxa influenced their geographical ranges of fossil distributions (Fig. 3).

Figure 4 illustrates that the larger indices of the latitudinal ranges and the geological durations are observed only in the smaller ammonitella diameters. The exceptions are those in the Carboniferous, Triassic, and Jurassic, in which such relationships are blurred. These tendencies in the analyses that reduce the influence by the geological durations imply that the hatchling size was one of the main controlling factors for geographical ranges in ammonoid habitats at a given geological point in time: ammonoids with smaller hatchling sizes tended to have broader geographical ranges of ammonoid habitats.

Most ammonoid hatchlings are thought to have had a planktic mode of life (Kulicki, 1974; Kulicki, 1979; Kulicki, 1996; Drushchits, Doguzhayeva & Mikhaylova, 1977; Tanabe, Fukuda & Obata, 1980; Tanabe et al., 2001; Tanabe, Landman & Yoshioka, 2003; Tanabe & Ohtsuka, 1985; Landman, Tanabe & Shigeta, 1996; Westermann, 1996; Rouget & Neige, 2001; De Baets, Landman & Tanabe, 2015). In modern cephalopods, hatchlings smaller than approximately 3 mm are considered to have a planktic habit in the post-embryonic stages (Wani, 2011; De Baets et al., 2012; De Baets, Landman & Tanabe, 2015; Villanueva et al., 2016), which also suggests a planktic mode of life in most ammonoids. A planktic mode of life at the post-embryonic stage is thought to have had a significant impact on their geographical ranges (Landman, Tanabe & Shigeta, 1996; Westermann, 1996; Tajika & Wani, 2011; Ikeda & Wani, 2012; Brayard & Escarguel, 2013; Yahada & Wani, 2013; De Baets, Landman & Tanabe, 2015; Zacaï et al., 2016). However, there has been little discussion of how the hatchling sizes are related to their geographical ranges. The analyses that reduce the influence of geological duration in this study imply that the hatchling sizes were of importance to the geographical ranges of ammonoid habitats at each geological point in time, similar to those in modern cephalopods. This similarity to modern cephalopods shows a common reproductive strategy in ancient and modern cephalopods: the planktic mode of life at the post-embryonic stages was important in order to achieve wide geographic ranges.

Taxonomic treatment of ammonoid genera could also affect the geographical range and geological duration of the examined genus depending on how the examined species are attributed to a given genus. For example, Maeda (1993) examined “four” species of “two” genera of Late Cretaceous ammonoids and suggested that the examined nominal species was one species (i.e., one genus), based on the stratigraphic occurrence, morphological variation, and shell ontogeny. Such integration and separation of a genus would influence the geographical range and geological duration, the averages of ammonitella diameters, which impact analyses such as this study.

### Comparison between different periods

The examination of the scatter diagrams for each period (Figs. 2–4) illustrates the characteristics of the relationship between the hatchling sizes and geographical ranges for each period. Scatter diagrams for the Permian and Cretaceous data demonstrate the same tendency as data for the aggregate Devonian–Cretaceous interval.

But scatter diagrams of the Devonian, Carboniferous, Triassic, and Jurassic (Figs. 2–4) demonstrate different tendencies. The ammonitella diameters in the Devonian tend to be larger than found in other periods and the maximum ammonitella diameter within the Devonian attained at least 6.0 mm (Table 1; De Baets, Landman & Tanabe, 2015). Several genera had ammonitella diameters larger than 3 mm, which is thought to be the approximated critical size for the planktic habit of the post-embryonic stage in modern cephalopods (Wani, 2011; De Baets et al., 2012; De Baets, Landman & Tanabe, 2015). De Baets, Landman & Tanabe (2015) mentioned a trend towards smaller ammonitella diameters from the Early to Late Devonian: the maximum ammonitella diameters were more than 3 mm in the Early Devonian, and less than 3 mm in the Late Devonian. This implies that ammonoids developed a reproductive strategy with smaller hatchling sizes during the Devonian, which possibly blurs the relationship between the latitudinal ranges, ammonitella diameters, and geological durations in the Devonian, at the examined temporal resolution (Fig. 3). Laptikhovsky, Nikolaeva & Rogov (2017) also concluded that the evolution of reproductive strategies in cephalopods in the geological past was marked by an increasing abundance of small-egged taxa, which agrees with the findings in this study.

The scatter diagram between the latitudinal ranges and geological durations in the Jurassic (Fig. 3C) shows a significant relationship. If only one long-ranged genus (*Phylloceras* of Phylloceratina) is excluded from the analyses, the scatter diagram showed no statistically significant correlation (*F* = 3.253 and *P* = 0.09). Such long-ranged genera are few in the examined data set and might indicate a possible problem with species determination; most ammonoid genera in the Triassic and Jurassic have shorter geological durations (Table 1; Table S1) (see Korn & De Baets, 2015, for a similar bias on the paleogeography of Devonian ammonoids). The larger indices of the geographical ranges and geological durations are observed not only in the smaller ammonitella diameters but also in the middle of the observed ranges of ammonitella diameters (Figs. 4C and 4D). Based on these values, the relationships between the hatchling diameters, geological duration, and geographical ranges in the Triassic and Jurassic are regarded as similar.

There were mass extinction events at the end of the Permian and Triassic when drastic taxonomic turnovers of ammonoid fauna occurred (e.g., House, 1988; Monnet, Brayard & Brosse, 2015; Brayard & Bucher, 2015; Longridge & Smith, 2015). In the Early Triassic and Early Jurassic, origination rates of ammonoids were high (Ceratitida originated in the Triassic and Lytoceratina and Ammonitina originated in the Jurassic; Neige & Rouget, 2015; Yacobucci, 2015; Longridge & Smith, 2015), which probably indicate radiations into open niche followed mass extinction events. At these times, ammonoids appear to have originated and resulted in an increased diversity rather than an increased geographical spread of each taxa (with the exception of some long-ranged taxa). This could blur the relationship between latitudinal ranges, geological durations, and ammonitella diameters in the Triassic and Jurassic (Figs. 2–4).

There was also a mass extinction event at the end of the Devonian, after which ammonoid diversity increased during the Carboniferous (Kullmann, 1981; Becker & Kullmann, 1996; Korn, Klug & Walton, 2015). As with the Triassic and Jurassic, originations following mass extinction events, those during the Carboniferous might influence the relationship between latitudinal ranges, geological durations, and ammonitella diameters. This is possibly seen in Fig. 4F where the larger indices tend to shift to the middle of the observed range of ammonitella diameters.

A more complete and detailed dataset of geographical range and geological duration with larger data size, together with the paleobiogeography of each period and morphological changes at the embryonic and post-embryonic stage, would allow us to analyze with more elaborate approaches and therefore to better understand the geological transition of ammonoid reproductive strategies during the Devonian–Cretaceous with finer geological resolution (i.e., at the stage level) and on a lower taxonomic level (i.e., at the species level).

##  Supplemental Information

10.7717/peerj.4108/supp-1 Table S1 Raw data of ammonitella diameter, latitudinal range, and geological duration

## Additional Information and Declarations

### Data Availability

The following information was supplied regarding data availability: The raw data is included as Table S1.
